# Association between tooth loss and orodigestive cancer mortality in an 80-year-old community-dwelling Japanese population: a 12-year prospective study

**DOI:** 10.1186/1471-2458-13-814

**Published:** 2013-09-08

**Authors:** Toshihiro Ansai, Yutaka Takata, Akihiro Yoshida, Inho Soh, Shuji Awano, Tomoko Hamasaki, Akira Sogame, Naoko Shimada

**Affiliations:** 1 Division of Community Oral Health Development, Kyushu Dental University, Kitakyushu, Japan; 2 Division of General Internal Medicine, Kyushu Dental University, Kitakyushu, Japan; 3 Division of Home Economics, Kyushu Women University, Kitakyushu, Japan; 4 Munakata and Onga Office for Health, Human Services and Environmental Issues, Munakata, Japan; 5 Kitakyushu Public Health and Welfare Bureau, Kitakyushu, Japan

**Keywords:** Dental care, Gastrointestinal cancer, Tooth loss

## Abstract

**Background:**

A growing body of evidence has indicated a possible association between oral and gastrointestinal (orodigestive) cancers and periodontal disease or tooth loss. However, the evidence remains contradictory. This study investigated whether tooth loss, which is indicative of poor oral health and a potential source of oral infections, is associated with death from orodigestive cancer.

**Methods:**

The study included 656 subjects in Fukuoka prefecture, Japan, who were 80 years old at baseline in 1998. All subjects underwent oral clinical examination and answered a questionnaire to determine their background characteristics. Cause of death over the 12-year follow-up was recorded from the registers at the Public Health Centers and classified according to the WHO International Classification of Diseases. Statistical analysis of associations was performed using Kaplan-Meier and Cox multivariate regression analyses.

**Results:**

A significant association was observed between tooth loss (continuous variable) and cancer death (hazard ratio (HR): 1.03, 95% confidence interval (CI): 1.00–1.07), after adjustment for potential confounders, including sex and smoking status. However, that association became insignificant in the fully adjusted model. On the other hand, tooth loss was significantly associated with orodigestive cancer (HR: 1.06, 95% CI: 1.01–1.13), even in the fully adjusted model including place of residence as a part of socioeconomic status.

**Conclusions:**

This study provides the first evidence in a prospective study in a Japanese population that tooth loss is associated with increased orodigestive cancer mortality, although the causality remains unclear.

## Background

A link between oral health status and all-cause mortality has been proposed, and there is evidence of a possible association between periodontal disease and atherosclerotic vascular disease [1]. In contrast, evidence of a relationship between oral health and cancer mortality is inconsistent [2-6]. For example, Michaud et al. [6] reported an association between all kinds of cancer and oral health status, including tooth loss and periodontal disease, in a prospective cohort study, and reported a significant association between tooth loss and esophageal cancer, but not between tooth loss and stomach cancer. However, two studies from China found an association between tooth loss and gastric cancer [3,4]. Putative mechanisms involved in the association between tooth loss and orodigestive cancers have been reviewed by Meurman and Bascones-Martinez [7], and infection and inflammation have been proposed as important risk factors. Orodigestive cancers were defined as cancers in the oral cavity and pharynx, esophagus, stomach, pancreas, liver, and colon, rectum or anus, as described in a recent report [8]. The association between poor oral health and increased risk of cancer at oral and pharyngeal sites was based on cross-sectional studies [9,10]. Thus, we aimed to perform a prospective study to examine the association between tooth loss and orodigestive cancer mortality in a cohort of elderly individuals from the general Japanese population over a 12-year period.

## Methods

### Study population

The Fukuoka 8020 survey, conducted between 1998 and 2010, was designed to examine the association between oral and systemic health conditions of the community-dwelling population at nine locations in Fukuoka Prefecture, as described previously [11]. Those 9 locations were selected randomly from urban, suburban, and rural communities to achieve a balance of living environments in terms of socio-demographic backgrounds, dietary habits, health behaviors, and available medical care. This study was designed as an investigation of a representative population of individuals residing in the eastern area of Kyushu Island who were born in 1917. The percentage of 80-year-old individuals living in the study locations was approximately 0.62% of all residents, which was similar to the percentage (0.64%) of 80-year-old individuals residing in all of Kyushu. All procedures were approved by the Human Investigations Committee of Kyushu Dental College, and all subjects provided written informed consent prior to participation. Three with a history of cancer at baseline were excluded. Of the 1282 80-year-old subjects included, 697 (54.4%; 277 male, 420 female) agreed to participate in the present study and completed a medical questionnaire, and also underwent physical, laboratory blood, and oral examinations. The mean proportion of elderly subjects in the communities was 24%, which was slightly higher than that in the total Japanese population (23%). The participants were followed up for 12 years after the baseline examination, with 41 (5.9%) lost to follow-up.

### Oral and systemic examinations

The baseline survey was performed in March 1998, and the participants took part in a personal interview performed by trained public health nurses, and answered a questionnaire containing 37 questions about oral and systemic health status, use of medical (or dental) services, personal hygiene, healthcare practices (including smoking habit), and medical conditions, as described before [11]. Dental health conditions including number of teeth were also examined by three dentists with comparable skills, as previously described [11]. Briefly, the examiners performed all oral examinations using criteria recommended by the World Health Organization [12]. To confirm inter-examiner reliability, duplicate examinations were conducted, and the agreement for dental health conditions (dental caries, missing, filling) was 92%.

### Endpoint ascertainment

Follow-up of the cohort was from the date of the baseline examination (in 1998) until June, 2010. Information on the survival of the subjects was collected from the registers at the Public Health Centers of each district included in the study, with cancer deaths coded by the International Classification of Diseases 10th Revision.

### Statistical analysis

Power analysis was performed using the software package G-Power. The statistical power of this study was found to be 86.9%, with sample sizes of 242 (alive during 12-year) for n1 and 414 (died during 12-year) for n2, an effect size of 0.25, and an α value of 0.05 set (two tailed t-test). Associations between baseline demographic and health-related characteristics and oral health status were examined with the Student *t*-test and Chi-squared test. Cox proportional hazards regression analysis was used to determine hazard ratios (HRs) and 95% confidence intervals (CIs) for mortality in relation to tooth loss and all deaths including cancer deaths. The quantitative variable (number of teeth) was converted into categorical variable (four groups): edentulous, 1–9 teeth, 10–19 teeth or 20 or more teeth, as described previously [13]. HRs were adjusted for sex and smoking status (never, past, current), and additionally for body mass index (BMI), fasting serum glucose, total cholesterol, and diastolic blood pressure. Comparisons of the survival rates among 4 groups based on number of teeth were also assessed by the method of Kaplan and Meier, followed by a log-rank test to assess the significance between survival curves. Statistical significance was indicated by two-sided P < 0.05. All statistical analyzes were performed using SPSS ver. 19 for Windows (SPSS, Chicago, IL, USA).

## Results

Of the 697 participants originally examined, 41 subjects (5.8%) had moved away during the 12 years, giving a follow-up rate of 94.2%. Of the remaining 656 participants, 414 (63.1%) had died by the end of follow-up in 2010. There were 71 deaths due to cancer, including lung cancer (n = 16), stomach cancer (n = 12), liver cancer (n = 13), colon cancer (n = 6), kidney and bladder cancer (n = 5), pancreatic cancer (n = 4), uterine cancer (n = 4), oropharyngeal cancer (n = 1), esophageal cancer (n = 1), ovarian cancer (n = 1), and other cancers (n = 11). Therefore, as described above, there were 37 deaths due to orodigestive cancer (19 males, 18 females). Demographic data and risk indicators at baseline in 1998 are presented in Table 1. Significantly more women than men were alive at the end of the study. Compared with survivors, subjects who died were more likely to be smokers (P < 0.001) and to be less physically active (P < 0.001). Both groups were similar regarding marital status, alcohol drinking habit, and self-rated health status. Mean BMI, total cholesterol, serum albumin, and fasting blood glucose, but not systolic blood pressure, were significantly lower at baseline in subjects who died than in survivors. There was a significantly higher number of missing teeth or proportion edentulous in those who died *versus* those who survived, indicating a poorer dental status in the subjects who died. Also, a significantly higher proportion of those who died were edentate.

**Table 1:** Baseline characteristics of the study population based on survival during the 12-year follow-up period

**Characteristic**	**Alive (n = 242)**	**Died (n = 414)**	**P value**
Female	178 (73.6)	211 (50.9)	< 0.001
Currently married	112 (46.3)	215 (51.9)	0.13
Place of residence			
Urban	65 (26.9)	85 (20.5)	< 0.001
Suburban	101 (41.7)	176 (42.5)	
Rural	76 (31.4)	153 (37.0)	
Medical examinations			
Serum total cholesterol	214.5 (35.3)	199.6 (38.2)	< 0.001
Fasting serum glucose	115.1 (40.8)	126.1 (57.5)	0.01
Serum albumin	4.3 (0.28)	4.2 (0.32)	< 0.001
Systolic blood pressure	150.9 (22.5)	149.7 (23.9)	0.53
Body mass index	23.2 (3.1)	22.4 (3.4)	0.005
Dental examinations			
Missing teeth	23.1 (9.2)	24.7 (8.5)	0.023
Number of teeth			
Edentulous	79 (32.6)	157 (37.9)	0.014
1–9 teeth	65 (26.9)	117 (28.3)	
10 – 19 teeth	56 (23.1)	87 (21.0)	
≥ 20 teeth	42 (17.4)	53 (12.8)	
Physical inactivity	3 (1.2)	43 (10.3)	< 0.001
High alcohol consumption	37 (15.3)	82 (19.8)	0.24
Smokers	7 (2.9)	67 (16.2)	< 0.001
Poor self-rated health	22 (9.0)	62 (14.9)	0.12

Data indicate the number of subjects (%) or mean (standard deviation).

Differences between groups were tested using a chi-square test for categorical variables and *t*-test for continuous variables.

**Figure 1:**
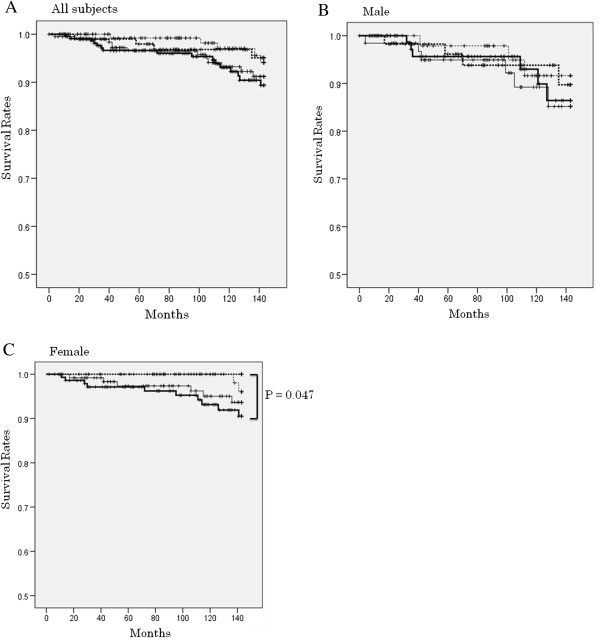
**Survival curves of the subjects who had not died due to orodigestive cancer during the 12-year follow-up period among the four groups divided by number of teeth for all subjects (n = 656) (A), male (n = 267) (B), and female (n = 389) (C).** 0 teeth,  1-9 teeth,  10-19 teeth,  ≥20 teeth

The survival curves of subjects who had not died due to orodigestive cancer during the 12-year follow-up period in the four groups divided by the number of remaining teeth in all subjects, as well as by sex are presented in Figure 1. The survival rate of all subjects was lowest in the edentulous subjects, though there was no significant difference among the four teeth groups (Figure 1A). As illustrated in Figure 1B, the survival rate for males was also lowest in the 1–9 teeth group, though there was no significant difference among the four teeth groups. In contrast, the survival rate for females was lowest in the edentulous groups, being significantly lower than that in the group with 20 teeth or more teeth (χ^2^ = 3.93, P = 0.047). Thus, a stronger association was found in females.

In multivariate Cox regression analyses, significant associations were observed between tooth loss (continuous variable) and all-cause mortality and cancer mortality (HR: 1.018, 95% CI: 1.005–1.03; HR: 1.03, 95% CI: 1.00–1.07, respectively), with adjustment for sex and smoking status. No significant associations of cardiovascular or pneumonia death and tooth loss were observed in the final model (HR: 1.018, 95% CI: 0.99–1.04; HR: 0.994, 95% CI: 0.97–1.02, respectively) (Table 2). Tooth loss was marginally significantly associated with stomach cancer (HR: 1.09, 95% CI: 0.98–1.22), and was significantly associated with orodigestive cancer (HR: 1.07, 95% CI: 1.02–1.13) with adjustment for sex and smoking status. Also, the observed association between tooth loss and orodigestive cancer remained significant (HR: 1.07, 95% CI: 1.02–1.13), even with full adjustment (sex, smoking status, BMI, fasting serum glucose, total cholesterol, serum albumin, physical activity) (Model 2 in Table 2). Furthermore, those associations remained significant (HR: 1.06, 95% CI: 1.01-1.13), when place of residence as a part of socioeconomic status was added in the final model (Model 3 in Table 2). Subjects with tooth loss had no significant risk of lung cancer, pancreatic cancer, colon cancer, and liver cancer, after extended adjustment in the final model (HR: 1.03, 95% CI: 0.96–1.10; HR: 0.96, 95% CI: 0.83–1.11; HR: 1.04, 95% CI: 0.92–1.18; HR: 1.07, 95% CI: 0.98–1.17, respectively).

**Table 2:** Number of missing teeth as continuous covariate and risk of mortality in the 12-year cohort study (1998–2010), with hazard ratios adjusted for potential confounders

**Cancer**	**Model 1 HR (95% CI)**	**P value**	**Model 2 HR (95% CI)**	**P value**	**Model 3 HR (95% CI)**	**P value**
Total cancer	1.033 (1.00–1.07)	0.048	1.035 (1.00–1.07)	0.047	1.032 (0.99–1.07)	0.068
Cancer site						
Lung	1.04 (0.97–1.10)	0.29	1.03 (0.97–1.10)	0.35	1.03 (0.96–1.10)	0.35
Stomach	1.10 (0.99–1.23)	0.08	1.09 (0.98–1.22)	0.11	1.09 (0.98–1.22)	0.18
Pancreas	1.01 (0.89–1.15)	0.83	0.98 (0.85–1.12)	0.78	0.96 (0.83–1.11)	0.60
Colon	1.03 (0.93–1.14)	0.59	1.03 (0.92–1.17)	0.55	1.04 (0.92–1.18)	0.52
Liver	1.07 (0.98–1.17)	0.12	1.07 (0.98–1.17)	0.12	1.07 (0.98–1.17)	0.14
Orodigestive	1.07 (1.02–1.13)	0.009	1.07 (1.02–1.13)	0.01	1.06 (1.01–1.13)	0.015
CVD	1.021 (0.99–1.05)	0.08	1.021 (0.99–1.05)	0.08	1.018 (0.99–1.04)	0.14
Pneumonia	1.002 (0.97–1.03)	0.91	0.993 (0.96–1.02)	0.62	0.994 (0.97–1.02)	0.67

Model 1: adjusted for sex and smoking status (never; past; current).

Model 2: additional adjustment for total cholesterol, serum albumin, fasting serum glucose, body mass index, physical activity.

Model 3: additional adjustment for place of residence.

*HR* Hazard ratio, *CI* Confidence interval, *NS* not significant, *CVD* Cardiovascular disease.

## Discussion

In this prospective study of a cohort of 80-year-old Japanese subjects, we have shown that number of teeth lost might be an independent predictor of both all-cause and cancer mortality. Even after extensive adjustment for recognized confounders, tooth loss was significantly associated with an increased risk of all-cause and cancer mortality, but not of cardiovascular disease (CVD) and pneumonia mortality. Interestingly, when we further assessed the association between tooth loss and site-specific cancer mortality, there were significant associations between tooth loss and orodigestive cancers, but not between tooth loss and lung cancer.

To our knowledge, previous longitudinal studies investigating the association between tooth loss and orodigestive cancer are limited to reports by three groups: Stolzenberg-Solomon et al. [2], Abnet et al. [3,4], and Michaud et al. [6]. The only investigation in an Asian population was by Abnet et al. [3]. Our study is the second Asian study and the first in a Japanese population, although a previous case–control study in 5,000 outpatients of a Japanese Cancer Center reported a positive association between tooth loss and the risk of head and neck, esophageal, and lung cancer [14]. In the study of Abnet et al. [3,4], there were two critical issues: 1) tooth loss was set as a dichotomous variable based on the median for the cohort, namely six teeth; and 2) only upper gastrointestinal (GI) cancers were investigated. In their study, upper GI cancer was defined as esophageal, gastric cardia, and non-cardia gastric cancers. In their report, tooth loss was significantly associated with increased risk of death from upper GI cancer (HR: 1.35, 95% CI: 1.14–1.59). Also, the risk of upper GI cancer associated with tooth loss was higher in male never-smokers than in male smokers (HR: 1.59, 95% CI: 1.03–2.45; HR: 1.39, 95% CI: 1.06–1.83, respectively). Results of multivariate analyses in a study in the United States [6] found no significant associations between tooth loss and morbidity due to stomach cancer, pancreatic cancer, or colorectal cancer (HR: 1.10, 95% CI: 0.56–2.16; HR: 0.91, 95% CI: 0.56–1.47; HR: 1.10, 95% CI: 0.87–1.37, respectively). However, in another study by the same researchers, a significant association between tooth loss and pancreatic cancer morbidity was observed (HR: 1.61, 95% CI: 1.13–2.31) [15]. In another Western country, Finland, there was a significant association between tooth loss and pancreas cancer morbidity (HR: 1.63, 95% CI: 1.09–2.46) [2]. Thus, these studies have reported conflicting results for the association between tooth loss and cancer morbidity or mortality.

On the other hand, we found no significant association between tooth loss and CVD mortality, which is inconsistent with the recent other study, as reported by Watt et al. [16]. This difference may be due to the difference in subject age (the mean age of their study was approximately 50 years old in that study).

One of the possible reasons underlying the different outcomes could be because of differences in the criteria used for measuring tooth loss. In most cases, a dichotomous variable was used. For example, Stolzenberg-Solomon et al. [2] used two categories, edentulism *vs.* 0–10 missing teeth, while Michaud et al. [6] used three categories of 0–16, 17–24, and 25–32 teeth. Those results indicate that cut-off values for number of teeth utilized there have not been standardized. Because of this disparity, we employed the number of missing teeth as a continuous variable in the present study, as it basically represents an accumulated burden of severe periodontal disease as the number increases. However, the association might not always be linear, as the association between CVD mortality and the number of missing teeth was shown to be non-linear in the study of Tu et al. [5]. A similar situation has been also been found in periodontal disease assessments. Periodontal disease is generally diagnosed by probing and its diagnosis of periodontal disease is not straightforward, thus it would be inappropriate to use for determination of the underlying disease status. As pointed out by Tu and Gilthorpe [17], an alternative method is to use the number of lost teeth, as tooth loss appears to be a better indicator than probing as a marker of lifetime oral health, and is less prone to measurement error. International standardization regarding evaluation by tooth loss or periodontal disease is required.

There are several limitations in the present study. First, the sample consisted largely of generally healthy elderly subjects, who might have been more eager and/or able to participate. Thus, our findings may indicate an association only in generally healthy elderly subjects. Second, our subjects in this study were all 80 years old at baseline, which is a very elderly population. It is possible that the mortality rates for both total and specific cancers could be underestimated because of a survivor effect. Thus, future investigations in a younger population will be necessary to confirm the validity of our results. Third, evaluation of the mortality risk for specific cancers was limited because of the small number of site-specific orodigestive cancer deaths.

## Conclusions

The results of this study showed a significant positive association between tooth loss and orodigestive cancer mortality risk, although the causal relationship remains unclear.

## Competing interests

The authors declare that they have no competing interests.

## Authors’ contributions

The epidemiological study was supervised by TA. TA, YT, AY, IS, SA, TH, AS, and NS participated in the epidemiological study. TA and YT participated in the design of the study and performed the statistical analysis. TA wrote the first draft of the manuscript. All authors read and approved the final version of the manuscript.

## Pre-publication history

The pre-publication history for this paper can be accessed here:

http://www.biomedcentral.com/1471-2458/13/814/prepub
